# Supplementary material for: Patients with Very High Risk of Cardiovascular Adverse Events during Carfilzomib Therapy: Prevention and Management of Events in a Single Center Experience
Source: Cancers (Basel). 2023 Feb 10;15(4):1149. doi: 10.3390/cancers15041149 (PMC9953901; doi:10.3390/cancers15041149)
Supplement: Supplementary file 1 [file cancers-15-01149-s001.zip › cancers-2160295-supplementary.pdf]

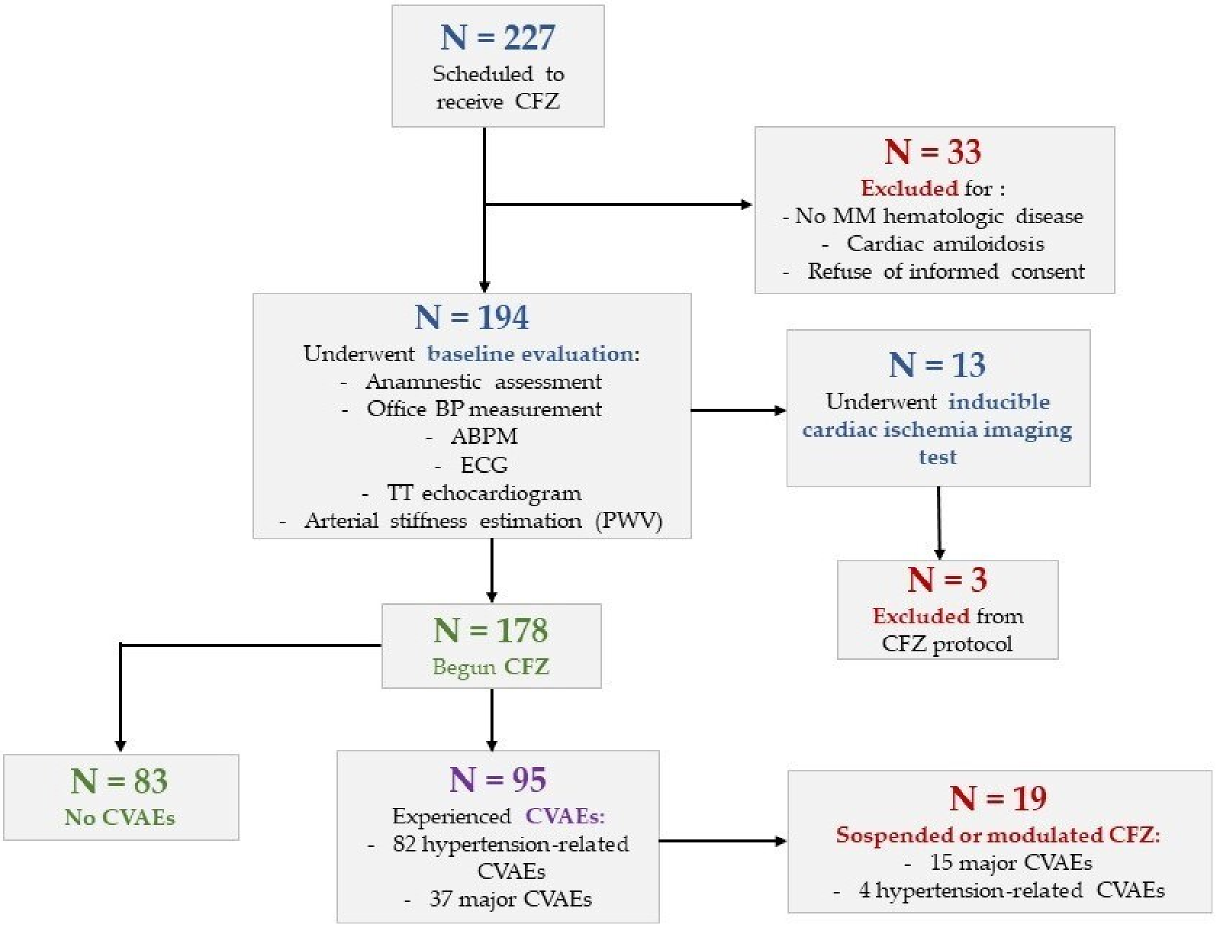

CFZ: Carfilzomib; MM: multiple myeloma; BP: blood pressure; ABPM ambulatory blood pressure measurement; ECG: electrocardiogram; TT: transthoracic; PWV: pulse wave velocity; CVAEs: cardiovascular adverse events.

Figure S1. Flowchart of the study population.
